# Treatment of talus osteochondral defects with arthroscopy debridement and bone marrow concentrate: a 6-month follow-up case report study

**DOI:** 10.1093/jscr/rjad365

**Published:** 2023-06-21

**Authors:** Konstantin Mitev

**Affiliations:** Orthopedics and Traumatology, Zan Mitrev Clinic, Skopje, Republic of North Macedonia

## Abstract

Talus osteochondral defects are a common cause of ankle pain and disability, and require prompt and effective treatment to prevent further damage and improve function. While surgical interventions, such as arthroscopy debridement and bone marrow concentrate therapy, have been used separately to treat these injuries, their combination may offer synergistic benefits. A 28-year-old male patient presented with a history of ankle pain and difficulty with weight-bearing activities. Post-operatively, the patient reported significant improvement in pain and function.

## INTRODUCTION

Talus osteochondral defects are a common cause of ankle pain and disability, and require prompt and effective treatment to prevent further damage and improve function [1]. While surgical interventions, such as arthroscopy debridement and bone marrow concentrate (BMC) therapy, have been used separately to treat these injuries, their combination may offer synergistic benefits [2]. This case report study aims to evaluate the efficacy of combining arthroscopy debridement and BMC therapy in the treatment of a patient with talus osteochondral defects, with a 6-month follow-up.

## CASE REPORT

**Figure 1:**
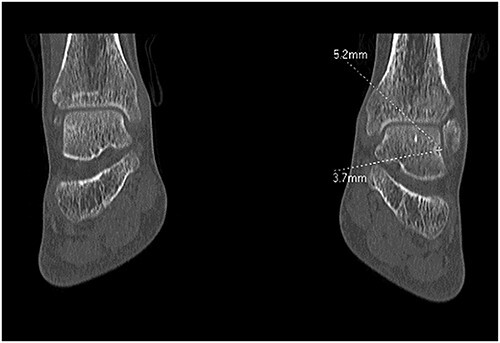
CT right ankle, arrow osteochondral lesion of the talus.

**Figure 2:**
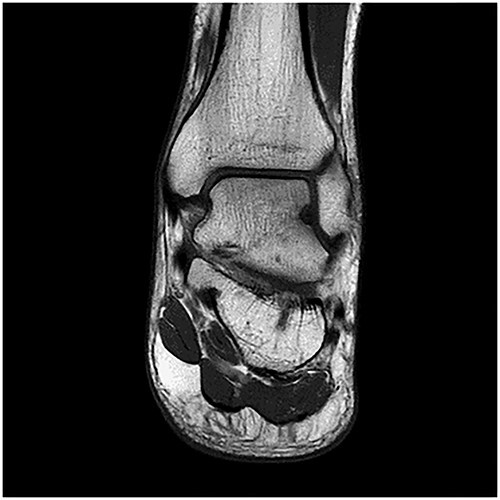
MRI ankle.

**Figure 3:**
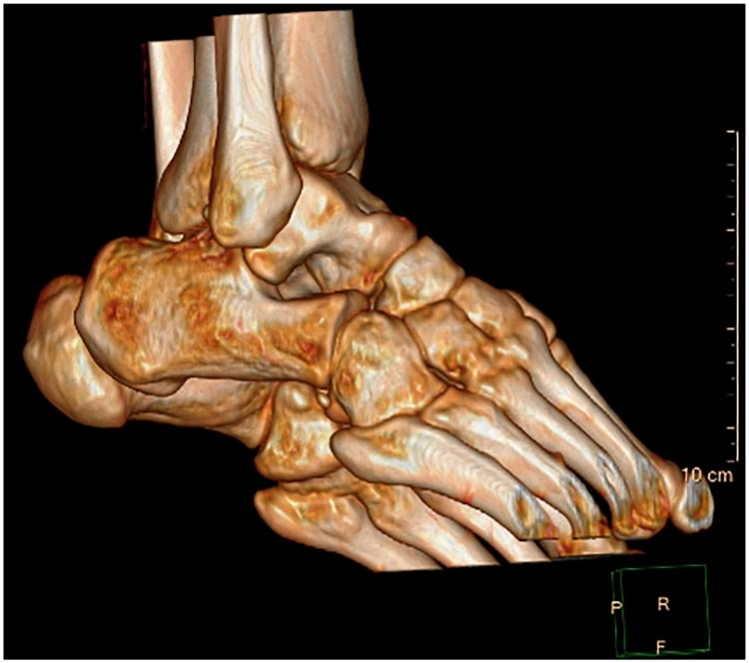
3D CT reconstruction.

A 28-year-old male patient presented with a history of ankle pain and difficulty with weight-bearing activities on the right side. Radiographic evaluation revealed a large osteochondral defect in the talus. (Figs 1–3).

**Figure 4:**
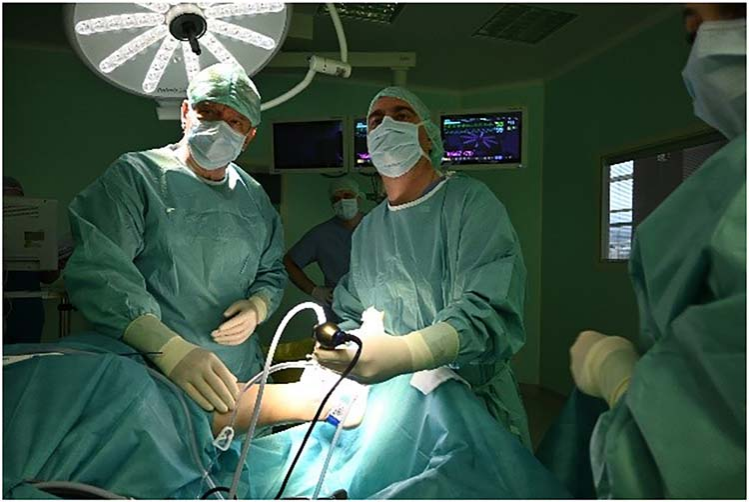
Arthroscopy of the ankle.

**Figure 5:**
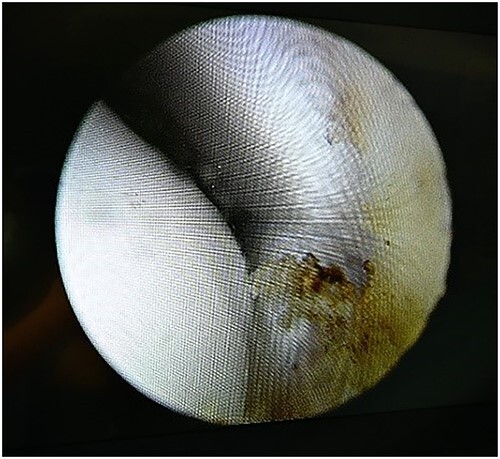
Arthroscopy view.

**Figure 6:**
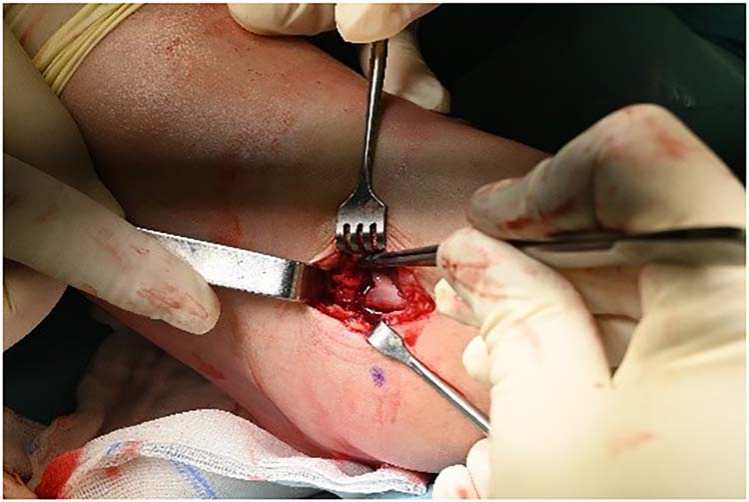
Mini open artrotomy.

**Figure 7:**
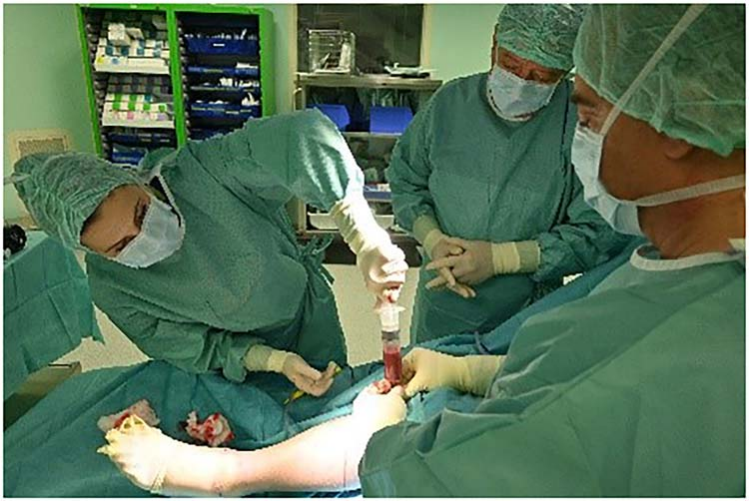
Bone marrow aspirate from proximal tibia.

**Figure 8:**
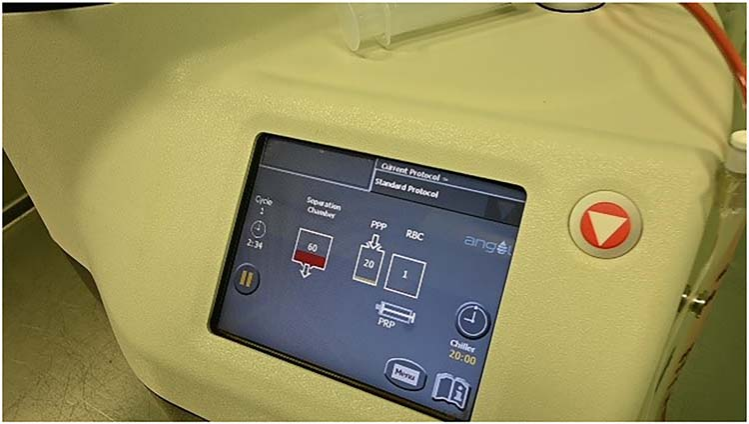
Monitoring of the bone marrow concentrate.

The patient underwent ankle arthroscopy (Fig. 4), mini open debridement of the affected area (Figs 5 and 6), followed by intra-articular injection of BMC derived from his own bone marrow. (Figs 7 and 8).

**Figure 9:**
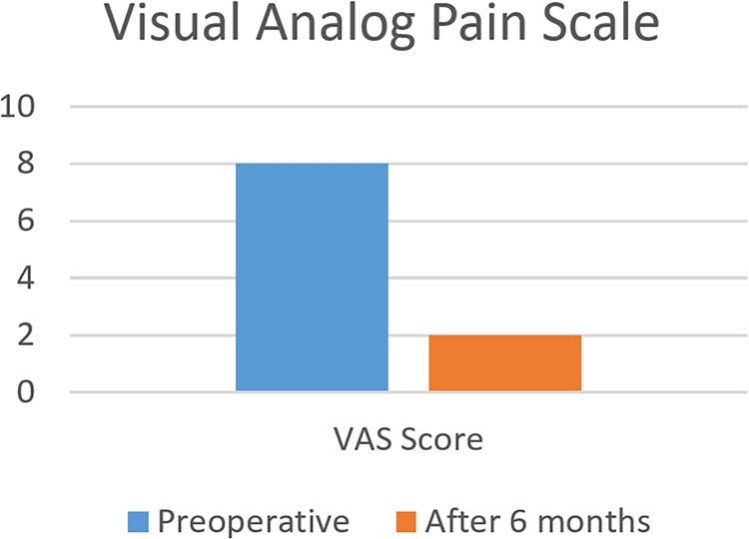
Pre and postoperative visual analog pain scale.

**Figure 10:**
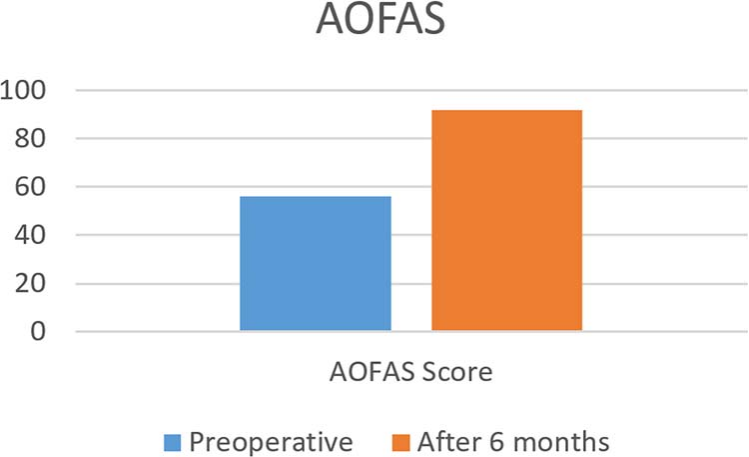
Pre and postoperative AOFAS.

Post-operatively, the patient reported significant improvement in pain and function. Clinical evaluation, including the American Orthopaedic Foot and Ankle Society (AOFAS) ankle-hindfoot score and the visual analog scale (VAS) for pain, was performed at 6 months after the procedure. The VAS score improved from 8 pre-operatively to 2 at 6 months, while the AOFAS score improved from 56 pre-operatively to 92 at 6 months (Figs 9 and 10).

No major complications were reported.

## DISCUSSION

This case report study supports the efficacy of combining arthroscopy debridement and BMC therapy in the treatment of talus osteochondral defects. The results suggest that this combination therapy may be a safe and effective alternative to traditional surgical options, offering significant improvement in pain and function for patients with talus osteochondral defects. Treating osteochondral lesions with subchondral drilling and microfracture are confirmed as reliable and effective techniques [3]. Newest studies suggest that concentrate bone marrow aspirate contains cytokines who are bioactive, similar as mesenchymal stem cell, which have ability for differentiation into chondrocytes [4]. Some of the research studies have shown that application of concentration of bone marrow aspirate can be effective in the repair of osteochondral defects [5]. Further randomized controlled trials with larger sample sizes and longer follow-up periods are needed to confirm these findings and to fully understand the potential benefits and limitations of this technique.

## CONCLUSION

The results of this case report study suggest that the combination of arthroscopy debridement and BMC therapy may be a promising option for the treatment of talus osteochondral defects. This approach may offer significant improvement in pain and function for patients with these injuries, with a low risk of major complications. Further research is needed to validate these findings and to fully understand the potential benefits and limitations of this technique.
